# A New Sensitive Sensor for Simultaneous Differential Pulse Voltammetric Determination of Codeine and Acetaminophen Using a Hydroquinone Derivative and Multiwall Carbon Nanotubes Carbon Paste Electrode

**DOI:** 10.1155/2015/783157

**Published:** 2015-04-06

**Authors:** Elahe Garazhian, M. Reza Shishehbore

**Affiliations:** Department of Chemistry, Yazd Branch, Islamic Azad University, P.O. Box 89195-155, Yazd, Iran

## Abstract

A new sensitive sensor was fabricated for simultaneous determination of codeine and acetaminophen based on 4-hydroxy-2-(triphenylphosphonio)phenolate (HTP) and multiwall carbon nanotubes paste electrode at trace levels. The sensitivity of codeine determination was deeply affected by spiking multiwall carbon nanotubes and a modifier in carbon paste. Electron transfer coefficient, *α*, catalytic electron rate constant, *k*, and the exchange current density, *j*_0_, for oxidation of codeine at the HTP-MWCNT-CPE were calculated using cyclic voltammetry. The calibration curve was linear over the range 0.2–844.7 *μ*M with two linear segments, and the detection limit of 0.063 *μ*M of codeine was obtained using differential pulse voltammetry. The modified electrode was separated codeine and acetaminophen signals by differential pulse voltammetry. The modified electrode was applied for the determination of codeine and acetaminophen in biological and pharmaceutical samples with satisfactory results.

## 1. Introduction

**Scheme 1:**
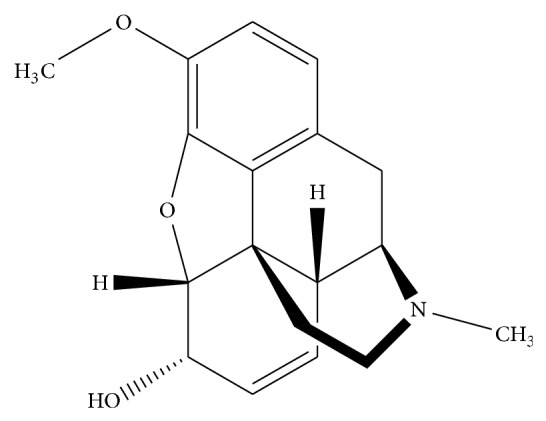
Molecular structure of codeine.

Codeine (3-methyl morphine, see Scheme 1 for molecular structure) is the second most predominant alkaloid in the opium poppy. For the first time, codeine was isolated and recognized while working on refined morphine extraction processes by Pierre Robiquet, the French chemist and pharmacist, in 1832. It has pharmacological and toxicological activity. Based on WHO reports, codeine is currently the most widely used opiate in the world. Also, it is one of the most commonly used drugs overall [1]. Since codeine can be used as recreational drug, a heroin addict may use codeine to ward off the effects of a withdrawal. Codeine and its metabolites can be determined in biological fluids to monitor therapy or screen drug abuse [1, 2]. Therefore, quantitative determination of codeine in biofluids can be attractive for clinical and forensic aims.

Various reports have been found for the determination of codeine in real samples with different matrices. They are including gas chromatography-mass spectrometry [3], micellar electrokinetic chromatography [4], high performance liquid chromatography-mass spectrometry [5], and capillary zone electrophoresis [6]. Along with the low detection limit, shortages such as setup cost and hard operation were found in application of chromatographic methods. Other methods are chemometrics assisted thermogravimetry analysis [7], flow injection analysis [8], and spectrofluorimetry [9]. In recent years, electroanalytical chemists have shown an interest in the determination of codeine, and various modified electrodes [10–17] have been constructed. Since electrochemical methods are of low cost, rapid, and simple, they have great importance in fields of environmental monitoring [18], medicine, and biotechnology [19] and different applications in industrial process control.

Acetaminophen (paracetamol, N-acetyl-*p*-aminophenol) is a widely used over-the-counter pain reliever and fever reducer. It was introduced by Harmon Northrop Morse in 1877. It is classified as a mild pain reliever that is commonly used for the relief of headaches and other minor aches and pains. It is approved for reducing fever in people of all ages. Also, it is used for the relief of pains associated with many parts of the body that is comparable to that of aspirin, while its anti-inflammatory effects are weaker. It is better tolerated than aspirin in patients in whom excessive gastric acid secretion or prolongation of bleeding time may be a concern. Untreated acetaminophen overdose results in a lengthy and painful illness. Signs and symptoms of acetaminophen toxicity may initially be absent or nonspecific symptoms. The process of dying from an overdose usually takes three to five days [20, 21]. In the last decade, numerous methods have been reported for the analysis of acetaminophen in pharmaceutical samples and biofluids. They are including colorimetry [22], spectrophotometry [23], NIR spectroscopy [24], capillary electrophoresis [25], microemulsion liquid chromatography [26], high performance liquid chromatography [27, 28], and electrochemical methods that are the most conventional methods [29–34].

Acetaminophen in combination with opioid analgesics like codeine can also be used in the management of more severe pain such as postsurgical pain and providing palliative care in advanced cancer patients and improving the efficacy for about 50% of patients [35]. Therefore, quantitative determination of codeine and acetaminophen individually or in the presence of both is important from different points of view. To the best of our knowledge, up to now, there was not found any report for the simultaneous determination of codeine and acetaminophen using electrochemical methods. In continuing of our research on the fabrication of 4-hydroxy-2-(triphenylphosphonio)phenolate (HTP)/multiwall carbon nanotubes modified carbon paste electrode (HTP-MWCNT-CPE) and its applications for the determination of different analytes [19, 36], we introduced a sensitive and selective sensor for the individual and simultaneous determination of codeine and acetaminophen using HTP-MWCNT-CPE for the electrocatalytic oxidation of codeine and the simultaneous determination of codeine and acetaminophen. The modified electrode shows excellent electrocatalytic activity toward codeine with low detection limit and wide linear dynamic range. Moreover, the modified electrode exhibits good ability for simultaneous determination of codeine and acetaminophen in serum and urine samples as biological and acetaminophen and acetaminophen-codeine tablet as pharmaceutical samples.

## 2. Experimental

### 2.1. Apparatus and Chemicals

All the electrochemical measurements were carried out using an Autolab potentiostat-galvanostat PGSTAT 30 (Eco Chemie, Netherlands) equipped with GPES 4.9 software. A three-electrode assembly was employed to the experiment in a 50 mL glass cell equipped with a HTP multiwall carbon nanotubes modified carbon paste electrode (HTP-MWCNT-CPE) as the working electrode, a graphite electrode as an auxiliary electrode, and a saturated calomel electrode, SCE, as a reference electrode. All of the potentials in the text are quoted versus this reference electrode. A personal computer was used for data storage and processing. A Metrohm 781 pH/mV meter was also used for pH measurements.

Codeine as codeine phosphate 1/2 H_2_O (Sigma, USA) and acetaminophen (Merck, Germany) with analytical grades were used as received. All of the solutions were freshly prepared using double-distilled water. Graphite fine powder (Fluka, Swiss) and paraffin oil (DC 350, Merck, density = 0.88 g cm^−3^) were used as binding agents for the graphite pastes. The multiwall carbon nanotube (MWCNT) with a diameter of 10–20 nm, length of 5–20 *μ*m, and purity of >95% was purchased from NanoLab Inc. (Brighton, MA). 4-Hydroxy-2-(triphenylphosphonio)phenolate (HTP) was synthesized as reported previously [37]. The buffer solutions were prepared from phosphoric acid and its salts.

### 2.2. Electrode Preparation

Modified carbon paste electrode was prepared in a conventional fashion by thoroughly hand-mixing of HTP (0.5 mg), MWCNT (1.0 mg), and graphite powder (100.0 mg) and in a mortar with a pestle. Paraffin was added to the mixture using a 5 mL syringe and mixed well to obtain a uniformly wetted paste. The HTP-MWCNT-CP electrode (HTP-MWCNT-CPE) was fabricated by packing the paste into the end of a Teflon rod (ca. 2 mm i.d. and 10 cm long) and leveled off with a spatula. Then, the electrical contact was made by inserting a copper wire into the Teflon rod at the end of the mixture. When necessary, a new surface was obtained by pushing an excess of paste out of the tube and polishing it on a white paper. HTP modified CPE (HTP-CPE) and multiwall carbon nanotubes modified carbon paste electrode (MWCNT-CPE) were made in the same way without adding MWCNT to the former and HTP to the carbon paste. Moreover, unmodified carbon paste electrode (CPE) was prepared by mixing graphite powder and paraffin to obtain a wetted paste and fabricated as discussed.

### 2.3. Sample Preparation

#### 2.3.1. Serum Sample Preparation

The human serum was collected from volunteers who had not taken codeine and acetaminophen. For purification, 5 mL of 10% CCl_3_CO_2_H was added to 5 mL of the serum, shaked, and centrifuged for 5 min at 5000 rpm. The sample was diluted 10 times by a 0.15 M phosphate buffer solution at pH 7.0 and was treated just after spiking of codeine and acetaminophen according to the given procedure by DPV technique.

#### 2.3.2. Urine Sample Preparation

Fresh human urine samples were obtained from different volunteers who had not taken codeine and acetaminophen. Each sample was diluted 30 times by a 0.15 M phosphate buffer solution at pH 7.0 after filtering using Whatman filter paper (number 1). The electrochemical determination of codeine was done after spiking suitable amounts of codeine using DPV technique.

#### 2.3.3. Pharmaceutical Samples Preparation

Five acetaminophen tablets (in dose of 325 mg and 500 mg) acetaminophen-codeine (325–10 mg) were powdered and mixed thoroughly. An amount corresponding to a tablet was weighed, dissolved with 10.0 mL of water, and sonicated for 3 min. The sample was filtered through a Whatman filter paper (number 1), transferred to a 25 mL volumetric flask, and diluted to the mark with phosphate buffer solution at pH 7.0. A suitable aliquot of the solution was used for analysis using the procedure. Also, the sample was spiked with different amounts of codeine and acetaminophen and quantified using DPV technique.

## 3. Results and Discussion

### 3.1. Electrocatalytic Characteristic of HTP-MWCNT-CPE toward Oxidation of Codeine

**Figure 1:**
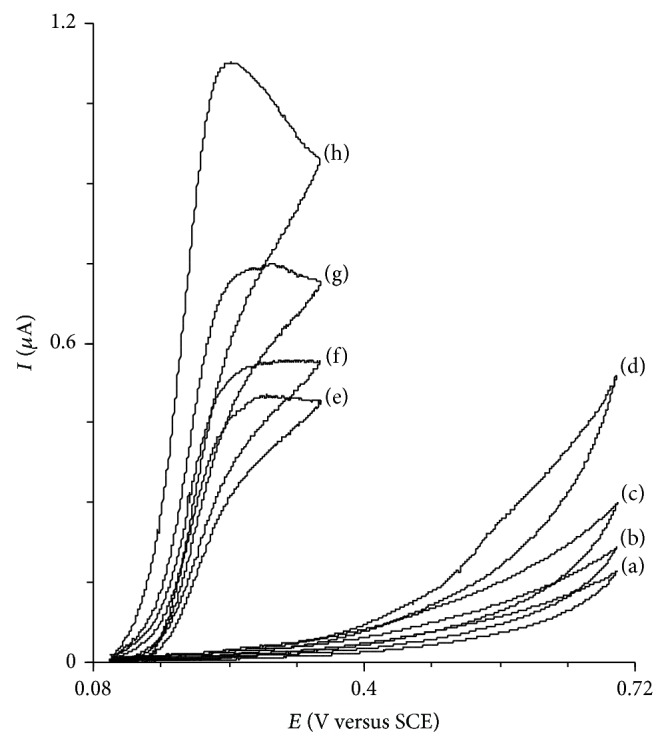
Cyclic voltammograms of (a) CPE, (c) MWCNT-CPE, (e) HTP-CPE and (g) HTP-MWCNT-CPE in a 0.15 M phosphate buffer solution (pH 7.0). (b) as (a), (d) as (c), (f) as (e) and (h) as (g) in presence of 0.11 mM of codeine. Scan rate: 25 mV s^−1^.

The activity of MWCNT and HTP as a modifier and the potential of electrocatalytic oxidation of codeine at the surface of different modified electrodes including MWCNT-CPE, HTP-CPE, and HTP-MWCNT-CPE was investigated by recording the cyclic voltammograms in the absence and presence of 0.11 mM of codeine solution and comparison of them with the same voltammograms was recorded at the CPE. The electrocatalytic activity is discussed in detail as follows: voltammograms of (a) and (b) of Figure 1 show the cyclic voltammograms of CPE in the absence and presence of codeine, respectively. Similarly, the cyclic voltammograms of MWCNT-CPE are recorded in the absence (voltammogram (c)) and presence (voltammogram (d)) of 0.11 mM of codeine. A comparison of the cyclic voltammograms of CPE (Figure 1, voltammograms (a) and (b)) and MWCNT-CPE (Figure 1 voltammograms (c) and (d)) in a 0.15 M phosphate buffer solution (pH 7.0) at the scan rate of 25 mV s^−1^ demonstrates the efficiency of MWCNT for codeine oxidation. As it can be seen, codeine oxidation at the MWCNT-CPE was performed at potential about 550 mV with anodic peak current 0.205 *μ*A. However, no anodic peak current was observed in other cases. Therefore, MWCNT can improve the sensitivity of the CPE for electrocatalytic oxidation of codeine.

Cyclic voltammograms of HTP-CPE in codeine-free electrolyte, 0.15 M of a phosphate buffer solution at pH 7.0 (voltammogram (e)), and 0.11 mM of codeine (voltammogram (f)) at the scan rate potential of 25 mV s^−1^ were recorded. As it can be seen, codeine oxidizes at 274 mV at HTP-CPE surface, while the anodic peak current for oxidation of codeine at MWCNT-CPE is about 550 mV. Moreover, the anodic peak current increased to 0.545 *μ*A. Thus, HTP caused the overpotential to decrease, 276 mV, and the sensitivity to increase more than twice. This is an expected behavior for a modifier. At the same experimental conditions, the cyclic voltammograms of HTP-MWCNT-CPE in the absence (voltammogram (g)) and presence of 0.11 mM of codeine (voltammogram (h)) were recorded. A comparison of the electrocatalytic oxidation peak current of codeine at HTP-CPE (voltammogram (f)) and HTP-MWCNT-CPE (voltammogram (h)) shows that spiking of MWCNT to HTP-CPE increases the current response from 0.545 *μ*A to 1.12 *μ*A. Therefore, combination of MWCNT and HTP improves the sensitivity and dramatically reduces the peak potential in regard to electrocatalytic oxidation of codeine. The dependence of sensitivity to percent of MWCNT in the composition of carbon paste was investigated in the range 0.8–1.1% with percent interval 0.1%. Since the maximum sensitivity was obtained at 0.1% of MWCNT in composition of carbon paste, the percent was used for fabrication of the modified electrode in all subsequent studies. Also, the effect of modifier amount was checked with using the different values of 0.4 to 0.6 mg of HTP with weight interval 0.1 mg in preparation of the modified carbon paste electrode (as described previously). The obtained results show that the difference in voltammetric response of modified carbon paste electrode containing 0.5 and 0.6 mg of HTP is almost the same and is higher than that containing 0.4 mg. Thus, 0.5 mg of HTP was used for preparation of the modified carbon paste electrode. The pH effect on the cyclic voltammetric peak current of codeine electrocatalytic oxidation at the modified electrode was investigated by a 0.15 M phosphate buffer solution in pH range 6.0–8.0 with pH interval 1.0 on various concentrations of codeine. Since the widest linear dynamic range was obtained in pH 7.0 (12.3–511.9 *μ*M) and the sensitivity at pH of 7.0 and 8.0 (0.0231 and 0.0248 *μ*A *μ*M^−1^) as the same as and greater than the sensitivity of pH 6.0 (0.0167 *μ*A *μ*M^−1^), pH 7.0 was selected for further studies.

**Figure 2:**
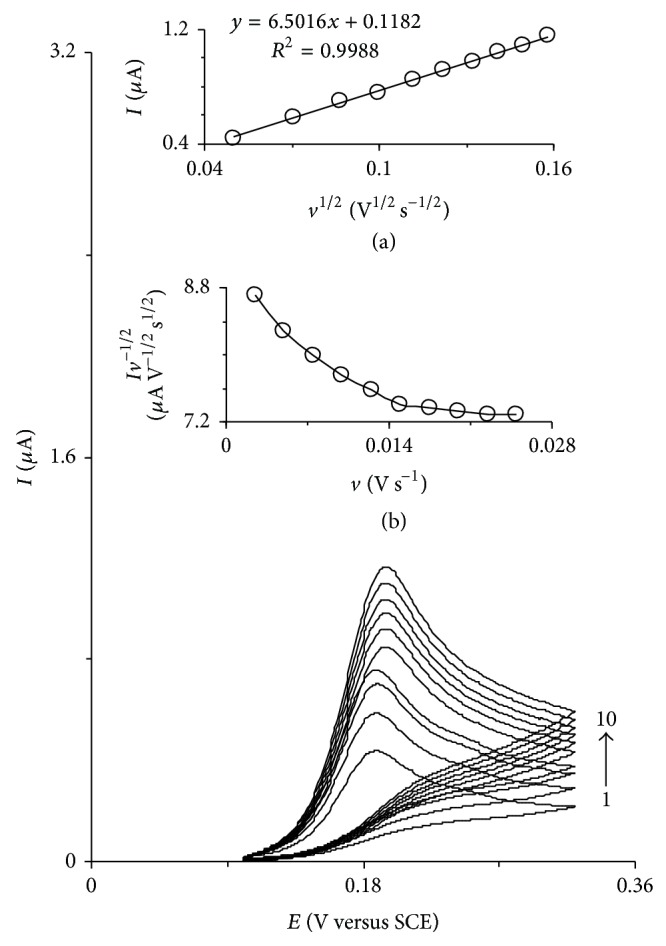
Cyclic voltammograms of HTP-MWCNT-CPE in a 0.15 M phosphate buffer solution (pH 7.0) containing 0.11 mM of codeine at different scan rates. The numbers of 1–10 correspond to scan rates of 2.5, 5, 7.5, 10, 12.5, 15, 17,5, 20, 22.5, and 25 mV s^−1^. Insets: (a) variation of the electrocatalytic peak currents versus the square root of scan rate and (b) variation of the scan rate normalized peak current (*I*_*p*_ *v* ^−1/2^) versus the scan rate.

Figure 2 shows the cyclic voltammograms of HTP-MWCNT-CPE in a 0.15 M phosphate buffer solution (pH 7.0) in the presence of 0.11 mM of codeine at different potential scan rates. Inset (a) of Figure 2 shows that the plot of the catalytic peak current versus the square root of the potential scan rate is linear, thus suggesting that at sufficient overpotential the reaction is diffusion-controlled. The slope of *I*_*P*_ versus *v* ^1/2^ plot is used for the determination of the number of electrons in the overall reaction. Based on the following equation for totally irreversible diffusion controlled processes [38]:

(1) $$\begin{array}{c} I_{p}=3.01\times 10^{-5}{n[\left(1-\alpha \right)n_{\alpha }]}^{1/2}AC_{b}D^{1/2}\nu^{1/2} \end{array}$$

and considering (1 − *α*)*n*_*α*_ = 0.65 (as calculated below), *D* = 1.35 × 10^−5^ cm^2^ s^−1^ (obtained by chronoamperometry), and *A* = 0.0314 cm^2^, the total number of electrons (*n*) corresponding to codeine oxidation is calculated as 2.10 ≈ 2. The calculated value for the electrocatalytic oxidation of codeine is in agreement with the values reported in the literature [11, 13, 15, 16]. Also, the plot of the scan rate normalized current (*I*_*p*_ *v* ^−1/2^) versus the scan rate (inset (b)) exhibits a characteristic shape typical of an EC_cat_ process [39]. These results confirm that the overall electrochemical oxidation of codeine at a modified electrode might be controlled by a cross-exchange process operating between the redox site of the HTP-MWCNT-CPE and codeine and also the diffusion of codeine.

For slow potential scan rates, *v*, and large catalytic rate constants, *k*′, Andrieux and Saveant developed a theoretical model for a heterogeneous catalytic process EC catalytic mechanism and derived a relationship between the peak current and the concentration of substrate [40]:

(2) $$\begin{array}{c} I_{\text{cat}}=0:496nFAC_{s}D^{1/2}v^{1/2}{\left(\frac{RT}{nF}\right)}^{1/2}, \end{array}$$

where *D* and *C*_*b*_ are the diffusion coefficient (cm^2^ s^−1^) and the bulk concentration (mol cm^−3^) of substrate (in this case, codeine), respectively. The other symbols have their usual meanings. Low values of heterogeneous rate constant (*k*′) result in a coefficient with values lower than 0.496. The value of this constant at low potential scan rates (2.5–25 mV s^−1^) was found to be 0.31 for a HTP-MWCNT-CPE with a geometric area, *A*, of 0.0314 cm^2^ and *D* = 1.35 × 10^−5^ cm^2^ s^−1^ in the presence of 0.01 mM of codeine. Using Figure 1 in the theoretical paper of Andrieux and Saveant and using 0.31 as the constant, the average value of  *k*′ = (7.53 ± 1.9) × 10^−3^ cm s^−1^ was obtained.

**Figure 3:**
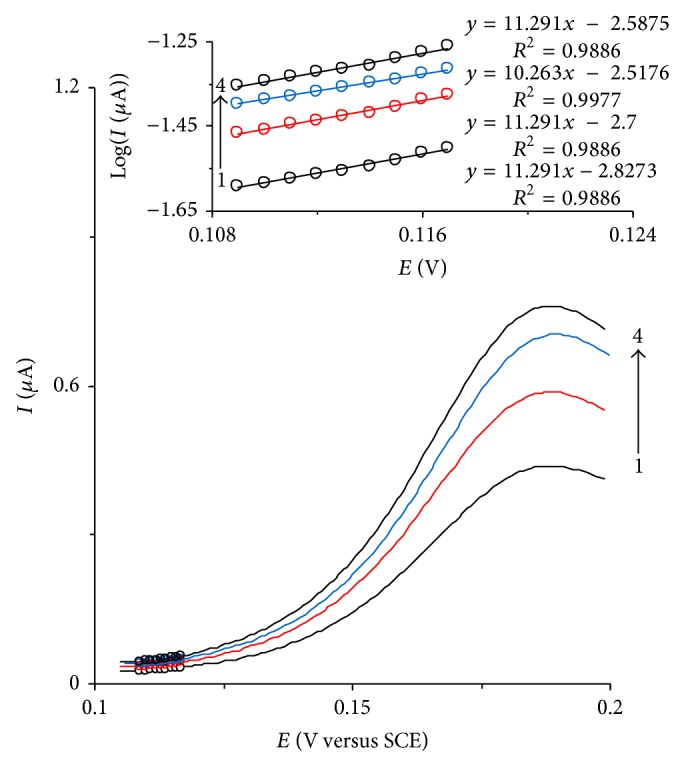
Cyclic voltammograms of HTP-MWCNT-CPE in a 0.15 M phosphate buffer solution (pH 7.0) containing 0.11 mM of codeine at scan rates 2.5, 5, 7.5, and 10 mV s^−1^. The circles show the part of cyclic voltammograms that was used for deriving Tafel plot. Inset shows the Tafel plots derived from the circled current-potential curve recorded at scan rates of 2.5, 5, 7.5, and 10 mV s^−1^.

Figure 3 shows the cyclic voltammograms of HTP-MWCNT-CPE in presence of 0.11 mM of codeine at scan rate potentials of 2.5, 5, 7.5 and 10 mV s^−1^. The data of the rising part of the voltammograms that characterized by circles were used for drawing the Tafel plots (inset of Figure 3). This part of the voltammogram is affected by the electron transfer kinetics between the substrate (in this case, codeine) and HTP-MWCNT-CPE, assuming the deprotonation of substrate as a sufficiently fast step [39]. The obtained value of Tafel slope for the oxidation of codeine (*b* = 2.3RT/(1 − *α*)*n*_*α*_ *F*) indicates that a one-electron transfer process is the rate-limiting step assuming a transfer coefficient of *α* = 0.35 ± 0.03. Also, the value of exchange current density, *j*_0_, was found from the intercept of the Tafel plot [39]. The value of *j*_0_ for codeine was obtained equal to 0.064 ± 0.024 *μ*A cm^−2^. The above results confirm that the catalytic oxidation mechanism of codeine at HTP-MWCNT-CPE surface is an *E*_*i*_ *C*_*i*_′ mechanism as shown in the following equations:
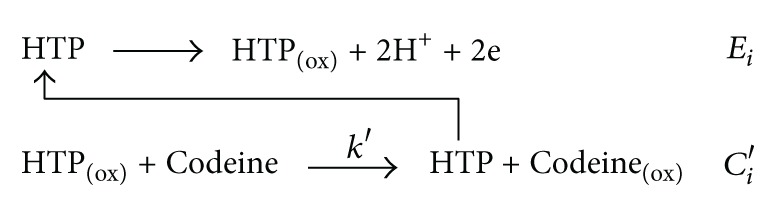
(3)


### 3.2. Chronoamperometric Measurements

**Figure 4:**
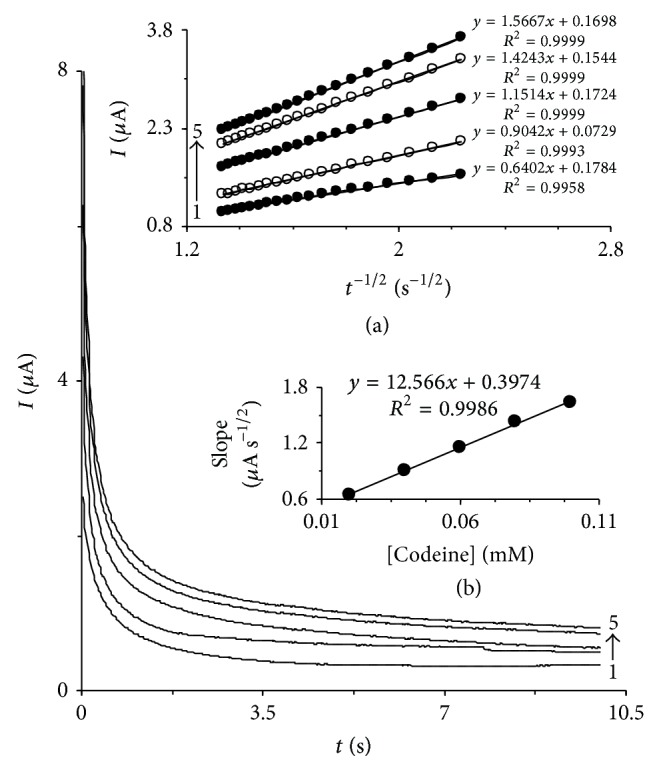
Chronoamperometric current responses at HTP-MWCNT-CPE surface in a 0.15 M phosphate buffer solution (pH 7.0), at a step potential of 235 mV, for different concentrations of codeine. The numbers of 1–5 correspond to 0.02, 0.04, 0.06, 0.08, and 0.1 mM of codeine. Insets: (a) plots of *I* versus *t* ^−1/2^ obtained from chronoamperograms and (b) plot of the slopes of the straight lines against the codeine concentrations.

Chronoamperometry technique was used for the determination of apparent diffusion coefficient of codeine at HTP-MWCNT-CPE surface under working experimental conditions. Chronoamperograms were obtained by setting the working electrode potential at 235 mV for various concentrations of codeine (Figure 4). For an electroactive material (in this case, codeine) with a diffusion coefficient of *D*_app_, the current of the electrochemical reaction (at a mass transport limited rate) is described by the Cottrell equation [39]:

(4) $$\begin{array}{c} I=nFAD^{1/2}Cs\pi^{-1/2}t^{-1/2}. \end{array}$$

Based on the Cottrell equation, the plot of *I* versus *t* ^−1/2^ will be linear. We have carried out such studies for various codeine concentrations at the modified electrode. Figure 4, inset (a), shows the experimental plots with the best straight lines for the different concentrations of codeine employed. The slopes of the resulting straight lines (Figure 4, inset (b)) were then plotted versus the concentration of codeine, from whose slope (12.566 *μ*A s^1/2 ^mM) and using the Cottrell equation, an apparent diffusion coefficient was calculated as 3.8 × 10^−6 ^cm^2^ s^−1^.

### 3.3. DPV Technique for Quantification of Codeine and Acetaminophen

**Figure 5:**
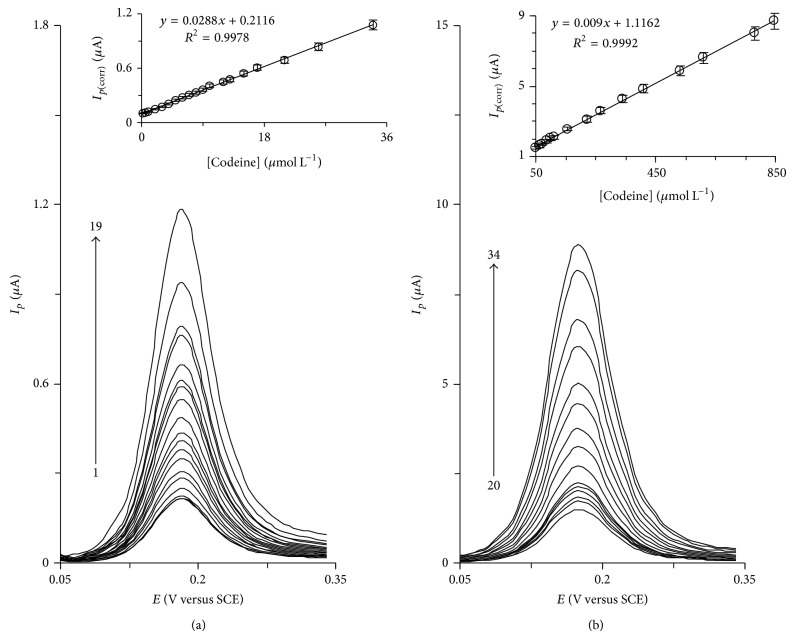
(a) and (b) show differential pulse voltammograms of HTP-MWCNT-CPE in a 0.15 M phosphate buffer solution (pH 7.0) containing different concentrations of codeine. The numbers of 1–19 and 20–34 correspond to 0.2–31.4 *μ*M and 34.1–844.7 *μ*M of codeine, respectively. Insets show the plots of electrocatalytic peak currents as a function of codeine concentrations in the ranges of 0.2–31.4 *μ*M and 34.1–844.7 *μ*M, respectively.

Since differential pulse voltammetry (DPV) has much higher current sensitivity and selectivity than cyclic voltammetry does, it was used to estimate the linear range, detection limit, and individual quantification of codeine and to simultaneously determine codeine and acetaminophen in various real samples. The effect of increasing the concentration of codeine in the range of 0.2–34.1 *μ*M on its voltammograms is presented in Figure 5(a) (in concentration range of 0.2–34.1 *μ*M) and Figure 5(b) (in concentration range of 34.1–844.7 *μ*M). Also, insets of the Figures 5(a) and 5(b) clearly show the plot of the corrected peak current, (*I*_*p*_)_corr_, versus codeine concentration that is constituted from two linear segments of 0.2–34.1 *μ*M and 34.1–844.7 *μ*M with slopes 0.0288 *μ*A *μ*M^−1^ and 0.009 *μ*A *μ*M^−1^, respectively. Corrected peak current is calculated from the difference between the observed current and the background current. The calibration curve, in first linear segment, was used to estimate the lower detection limit at the HTP-MWCNT-CPE surface. The detection limit, *C*_*m*_, was obtained to be 0.063 *μ*M of codeine by the equation *C*_*m*_ = 3*s*_*b*_/*m*, where *s*_*b*_ is the standard deviation of the blank response and *m* is the slope of the calibration curve (0.0288 *μ*A *μ*M^−1^). The average voltammetric peak current and the precision estimated in terms of the coefficient of variation for repeated measurements (*n* = 16) of 5.0 *μ*M of codeine at the proposed modified electrode were 0.34 ± 0.01 and 2.83%, respectively. This coefficient of variation value indicates that HTP-MWCNT-CPE is stable and does not undergo surface fouling during voltammetric measurements. This also demonstrates the fact that the results obtained at HTP-MWCNT-CPE are repeatable.  Table 1 gives the comparison of some of the analytical parameters obtained for codeine in this study with those previously reported by others. As it can be seen, the constructed modified electrode is superior in some cases, as compared to the previously reported modified electrodes.

**Figure 6:**
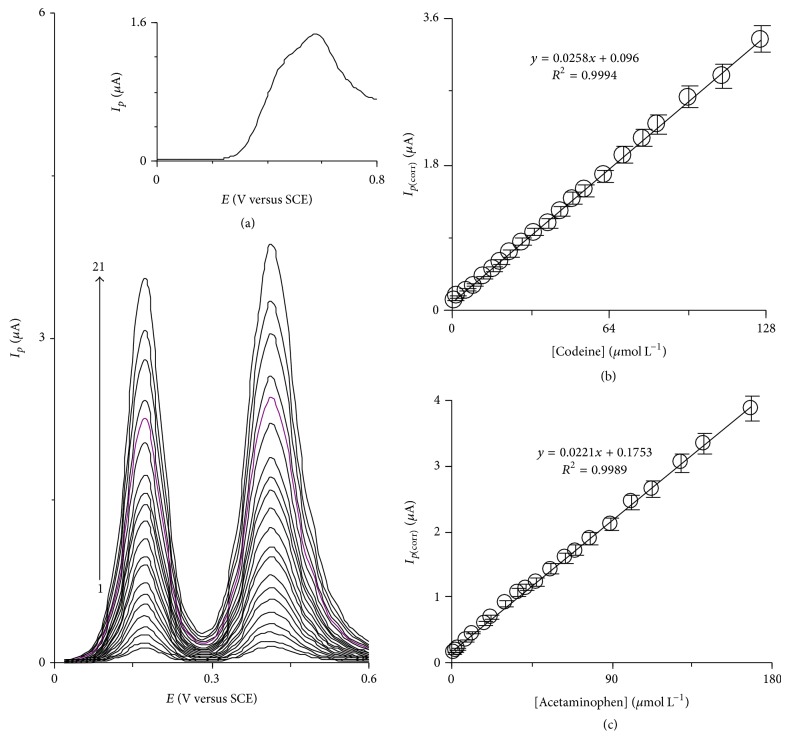
(a) Differential pulse voltammograms of HTP-MWCNT-CPE in a 0.15 M phosphate buffer solution (pH 7.0) containing different concentrations of codeine and acetaminophen. The numbers 1–21 correspond to 1.3–168.2 *μ*M of codeine and 0.95–125.8 *μ*M of acetaminophen. Inset shows differential pulse voltammogram of a mixed solution of 50.0 *μ*M of codeine and 110.0 *μ*M of acetaminophen at CPE. (b) and (c) show the plot of the corrected electrochemical peak currents as a function of codeine and acetaminophen concentrations in the range 1.3–168.2 *μ*M and 0.95–125.8 *μ*M, respectively.

The utility of the modified electrode was studied for the simultaneous determination of codeine and acetaminophen by simultaneously changing the concentrations of codeine and acetaminophen. The voltammetric responses show that the simultaneous determination of codeine and acetaminophen with two anodic peaks at potentials of 170 and 410 mV, corresponding to the oxidation of codeine and acetaminophen, is possible at HTP-MWCNT-CPE (Figure 6(a)). Figure 6(b) shows that the plot of corrected anodic peak current versus codeine concentration is linear in concentration range of 1.3–168.2 *μ*M. Also, Figure 6(c) demonstrates the linear correlation between corrected anodic peak current and acetaminophen concentration in concentration range 0.95–125.8 *μ*M while MWCNT-CPE could not separate the voltammograms of the mixed solution of 50.0 *μ*M of codeine and 110.0 *μ*M of acetaminophen (inset of Figure 6(a)). Also, the sameness of sensitivities of HTP-MWCNT-CPE to codeine in the absence (Figure 5(a); 0.0288 *μ*A *μ*M^−1^) and presence (Figure 6(b); 0.0258 *μ*A *μ*M^−1^) of acetaminophen indicates that the voltammetric responses of codeine and acetaminophen at HTP-MWCNT-CPE surface are independent of each other. Therefore, individual or simultaneous determination of codeine and acetaminophen is possible without any cross interferences.

### 3.4. Interference Study

Applicability and selectivity of the developed procedure were also evaluated by studying the effect of some common species that often accompany codeine in real samples. The study was performed by analyzing of 40.0 *μ*M of codeine in 0.15 M phosphate buffer solution (pH 7.0) containing concomitant species at different concentrations. The tolerance limit of coexisting species is defined as the largest amount that making variation of less than 5% in the recovery of the analyte. The results, given in Table 2, show that morphine and ascorbic acid have seriously interfering effect on the determination of codeine.

### 3.5. Real Sample Analysis

The evaluation of reliability and applicability of the developed procedure makes potential usefulness for quantitative determination of codeine and acetaminophen in samples with different matrices. Human serum and urine samples were selected as biological samples. After sample preparation as discussed in Sections 2.3.1 and 2.3.2, they were spiked with different amounts of codeine and acetaminophen. Using the developed procedure and calibration curves (Figures 6(b) and 6(c)), quantitation of the samples was done and the results are listed in Table 3. The relative standard deviations (RSD%) and the recovery rates of the spiked samples were acceptable and confirmed the applicability of the modified electrode for the determination of codeine and acetaminophen.

Also, acetaminophen tablets (in dosage of 325 and 500 mg) and acetaminophen-codeine tablets were used as pharmaceutical samples. Sample preparation was done as previously discussed in Section 2.3.3, and DPVs were recorded to estimate the codeine and acetaminophen concentrations using the calibration curves (Figures 6(b) and 6(c)). The results are summarized in Table 4. As it can be seen, the total values obtained for both analytes are in agreement with those registered in the label of the pharmaceutical inhalation products. A statistical test (*t*-test) was used to confirm the precision of the proposed method. The data in Table 3 suggest that the experimental values have a noticeable difference from the critical *t* value. The results imply that there is no evidence of systematic error in codeine and acetaminophen determination at the proposed modified electrode surface. Thus, HTP-MWCNT-CPE can be efficiently used for the simultaneous determination of codeine and acetaminophen in biological and pharmaceutical samples.

## 4. Conclusions

This study has demonstrated that the HTP-MWCNT-CPE can be applied for the quantitative determination of codeine. The results show that the combination of MWCNT and HTP improved the electrocatalytic oxidation characteristic of codeine. The mechanism of *E*_*i*_ *C*_*i*_′ was obtained by cyclic voltammetry. The DPV technique was used for plotting the calibration curve. The modified electrode not only improves the electrochemical catalytic oxidation of codeine, but also resolves the overlapping anodic peaks of codeine and acetaminophen as two well-distinguished anodic peaks. The analytical applicability of the introduced electrode was evaluated by applying it for the determination of codeine and acetaminophen in drug formulation and biological samples. Technical simplicity and possibility of rapid preparation, good reproducibility, stability, low detection limit, and wide linear concentration ranges for the determination of codeine and acetaminophen are the great advantages of this modified electrode. Quantitative determination of important biological compounds and drugs using the modifier can be considered for future trends.

## Figures and Tables

**Table 1:** Comparison of the efficiency of different modified electrodes for the determination of codeine.

Electrode name	Detection method	Modifier	Linear range (*µ*M)	Detection limit (*µ*M)	Sensitivity (*µ*A *µ*M^−1^)	Real sample	Reference
SWCNT/CCE^a^	DPV^h^	SWCNT	0.2–230.0	0.11	0.1959	Pharmaceutical samples, soft drinks, and urine	[10]

BBDE^b^	Amperometry	—	26.72–133.63	0.45	1.79	Pharmaceutical samples	[11]

GR-NF/GCE^c^	SWV^i^		0.05–30.0	0.015	16.20	Pharmaceutical samples and urine	[13]

DNA/MWCNT-PDDA/PGE^d^	DPV		0.17–133.6	0.14	2.6508	Drug formulations, urine, and plasma	[14]

BDDFE^e^	DPV	—	0.1–60.0	0.08	0.155	Human urine	[15]

PB/Pd-Al E^f^	DPV	PB	2.0–30.0	0.8	0.078	Synthetic sample	[16]

HTP-MWCNT-CPE^g^	DPV	HTP	0.2–34.1 34.1–844.7	0.063	0.0288 0.009	Pharmaceutical formulations, urine, and plasma	This work

^a^SWCNT/CCE: single-walled carbon nanotubes (SWCNT) carbon-ceramic electrode; ^b^BBDE: bare boron-doped diamond electrode; ^c^GR-NF: graphene-nafion film glassy carbon electrode; ^d^DNA/MWCNT-PDDA/PGE: DNA/multiwalled carbon nanotubes-poly(diallyldimethylammonium chloride)/pencil graphite electrode; ^e^BDDF electrode: boron-doped diamond film electrode; ^f^PB/Pd-Al E: prussian blue (PB) film modified-palladized aluminum electrode; ^g^4-hydroxy-2-(triphenylphosphonio)phenolate (HTP)-multiwall carbon nanotubes carbon paste electrode; ^h^DPV: differential pulse voltammetry; ^i^SWV; square wave voltammetry.

**Table 2:** Interference study of some species for the determination of 40.0 *µ*M of codeine at HTP-MWCNT-CPE.

Foreign species	Molar ratio (foreign species/codeine)
Na^+^, K^+^, Ca^2+^, Cl^−^, and NO_3_^−^	1000
Urea	695
Uric acid	675
Methadone, Tramadol	310
Morphine	5
Ascorbic acid	1

**Table 3:** Determination of codeine in human urine and serum samples using HTP-MWCNT-CPE.

Sample	Added (*μ*mol L^−1^)		Found^a^ (*μ*mol L^−1^)		RSD (%)		Recovery (%)	
	COD	AC	COD	AC	COD	AC	COD	AC
Serum	—	—	<DL	<DL	—	—	—	—
	10.0	20.0	10.1 ± 0.1	19.9 ± 0.2	1.1	1.0	100.8	99.6
	20.0	20.0	19.9 ± 0.2	20.2 ± 0.2	1.0	1.0	99.4	101.0
	40.0	60.0	40.2 ± 0.4	59.9 ± 0.6	1.0	1.0	100.5	99.8

Urine	—	—	<DL	<DL	—	—	—	—
	10.0	20.0	9.9 ± 0.1	20.2 ± 0.2	1.1	1.0	99.1	100.9
	20.0	20.0	20.1 ± 0.2	20.1 ± 0.2	1.0	1.0	100.6	100.6
	40.0	60.0	40.2 ± 0.4	59.9 ± 0.6	1.0	1.0	100.6	99.8

^a^Mean ± standard deviation (*n* = 4).

**Table 4:** Determination of codeine in pharmaceutical samples using HTP-MWCNT-CPE.

Sample	Added (mg)		Found^a^ (mg)		RSD (%)		Recovery (%)		Labelled value		Statistical *t*-test^b^		Company/batch number
	COD	AC	COD	AC	COD	AC	COD	AC	COD	AC	COD	AC	
AC tablet	—	—	—	324.2 ± 3.3	—	1.0	—	99.7	—	325.0	—	0.5	Jalinous/92046
	10.0	—	9.9 ± 0.1	324.2 ± 3.3	1.0	1.0	99.1	—	—	—	—	—	
	15.0	10.0	15.1 ± 0.2	335.2 ± 3.4	1.0	1.0	100.7	100.0	—	—	—	—	

AC tablet	—	—	—	504.1 ± 5.0	—	—	—	100.8	—	500.0	—	1.6	Soha/91025
	10.0	—	10.1 ± 0.1	503.0 ± 5.0	1.1	1.0	101.2	—	—	—	—	—	
	15.0	25.0	14.9 ± 0.2	526.1 ± 5.2	1.0	1.0	99.2	100.2	—	—	—	—	

AC-COD tablet	—	—	—	—	—	—	—	—	10	300.0	—	—	Pharmachemie/071
	—	—	10.1 ± 0.1	298.9 ± 3.1	1.1	1.0	100.8	99.6	—	—	1.4	0.7	
	5.0	—	14.9 ± 0.2	298.1 ± 3.2	1.0	1.1	99.4	—	—	—	—	—	
	—	5.0	9.8 ± 0.1	306.0 ± 3.3	1.2	1.0	98.2	100.3	—	—	—	—	
	5.0	5.0	15.4 ± 0.2	304.1 ± 3.2	1.0	1.0	102.4	99.7	—	—	—	—	

^a^Mean ± standard deviation (*n* = 4).

^b^Tabulated value of *t* at confidence limit of 95% and three degrees of freedom, 3.18.
